# Paeoniflorin drives the immunomodulatory effects of mesenchymal stem cells by regulating Th1/Th2 cytokines in oral lichen planus

**DOI:** 10.1038/s41598-022-23158-0

**Published:** 2022-11-04

**Authors:** Zhihui Zhang, Yan Zhang, Zhongfang Zhao, Pei Li, Danyang Chen, Wei Wang, Ying Han, Shiqi Zou, Xin Jin, Jianling Zhao, Hongwei Liu, Xiao Wang, Weili Zhu

**Affiliations:** 1 Stomatology Department, Peking University Third Hospital, Beijing, 100191 China; 2 Department of Stomatology, Peking University Third Hospital Yanqing Hospital, Beijing, China; 3 Department of Periodontology, School/Hospital of Stomatology, Lanzhou University, Lanzhou, Gansu Province China; 4 Department of Pediatric Dentistry, School and Hospital of Stomatology, Sun Yat-Sen University, Guangzhou, Guangdong Province China; 5 Dermatology Department, Peking University First Hospital, Beijing, China; 6 Oral Medical Center, Shenzhen People’s Hospital, Shen Zhen, Guangdong Province China; 7 Department of Oral Medicine, Peking University School and Hospital of Stomatology, Beijing, China; 8 Department of Stomatology, China-Japan Friendship Hospital, Beijing, China; 9 Stomatological Experimental Center, Dental Hospital Affiliated to Jiamusi University, Jiamusi, Heilongjiang Province China; 10 National Institute on Drug Dependence, Peking University and Beijing Key Laboratory of Drug Dependence Research, Peking University, Beijing, 100191 China

**Keywords:** Diseases, Oral diseases, Mucositis

## Abstract

Lichen planus (LP) is a chronic inflammatory disease. Oral lichen planus (OLP) mainly appears as oral mucosal reticular or ulcerative lesions with an unknown etiology. We aimed to explore the immunomodulatory effect of paeoniflorin (PF) in mesenchymal stem cells (MSCs) and the potential involvement of Th1/Th2 cytokines in OLP. The effects of paeoniflorin on the proliferation and migration of MSCs were detected by Cell Counting Kit-8 (CCK8) and Transwell assays. MSCs were subjected to osteogenic, adipogenic and neurogenic induction followed by Alizarin red, oil red O, real-time PCR and immunofluorescence assays. We found that paeoniflorin promoted the proliferation, migration and multilineage differentiation of MSCs from OLP lesions (OLP-MSCs) in vitro. Paeoniflorin pretreatment increased the inhibitory effect of OLP-MSCs on peripheral blood mononuclear cells. Furthermore, paeoniflorin-pretreated OLP-MSCs simultaneously decreased Th1 cytokine levels and increased Th2 cytokine levels in T lymphocyte cocultures. Finally, paeoniflorin-pretreated OLP-MSCs also promoted the Th1/Th2 balance both in vitro and in the serum of mice that received skin allografts. In conclusion, paeoniflorin enhanced MSC immunomodulation and changed the inflammatory microenvironment via T lymphocytes, suggesting that the improvement of OLP-MSCs is a promising therapeutic approach for OLP.

## Introduction

Lichen planus (LP) is a common chronic inflammatory disease that frequently involves the skin and mucous membranes. Oral lichen planus (OLP) mainly appears as oral mucosal reticular or ulcerative lesions^1^ and affects up to 4% of the general population worldwide, with a malignant transformation rate of approximately 5%^2–4^. The etiology of OLP is still unclear; however, immunological processes are believed to play critical roles^5^. It is well known that cell-mediated immune responses against antigens of the basal epithelial keratinocytes occur in OLP and involve lymphocyte–epithelium interactions^1,6^. Specifically, CD4 + cells are observed mainly in the lamina propria, with occasional T helper 1 (Th1) immune responses close to basal keratinocytes that may promote CD8 + cytotoxic T-cell activity in OLP. IFN-γ, TNF-α, IL-6 and GM-SF are released and induce a local inflammatory response, thus further aggravating tissue damage^7^. Furthermore, the imbalance of Th1/Th2 cytokine distribution is an important factor affecting the occurrence and pathological progression of OLP^8^. Therefore, deeply exploring the pathogenic factors of OLP and developing effective treatments on the basis of etiology is of great value for the clinical treatment and pathological understanding of this disease.

Mesenchymal stem cells (MSCs) are important members of the adult stem cell family and can be isolated from the bone marrow^9^, cord blood^10^, adipose tissue^11^, placenta^12^, dental pulp^13^, skin^14^ and tonsils^15^. MSCs have been demonstrated to have multilineage differentiation and immunomodulation capacities after recruitment and migration to sites of inflammation or injury^16^. In addition, MSCs suppress the activation, maturation and proliferation of innate or adaptive immune cells^17^. It has also been found that MSCs show great potential for modulating chronic inflammation and enhancing tissue regeneration in skin and other diseases^18,19^. MSCs have been proven to be effective in the treatment of immune- and inflammation-mediated diseases, such as graft-versus-host disease (GVHD)^20^, osteoarthritis (OA)^21^, and inflammatory bowel disease (IBD)^22^. LP and scleroderma-like lesions are the most common manifestations of chronic GVHD. Although it has been proposed that MSCs can be utilized to treat OLP patients via systemic infusion^23^, there is no direct evidence on the use of MSCS to treat OLP. Given the considerable therapeutic effects of MSCs on the inflammatory response, the exact mechanisms underlying their immunoregulatory activities need further clarification. Hence, a better understanding of OLP-MSCs extracted from inflammatory tissue and investigation of ways to improve the potential of MSCs may offer effective therapeutic strategies to achieve positive outcomes in patients with inflammatory diseases.

Paeoniflorin is an anti-inflammatory and immunomodulatory compound that is extracted from the roots of *Paeonia lactiflora* Pall and has a long history in traditional Chinese medicine^24^. It is the main bioactive component of total glucosides from paeony (TGP), which is a safe and effective systemic treatment for OLP^25^. Recently, numerous studies have confirmed the immunomodulatory effects of paeoniflorin^26–28^. For example, during the chronic inflammatory process, paeoniflorin inhibits the proliferation of fibroblast-like synoviocytes by suppressing G-protein-coupled receptor kinase 2^29^. Although MSCs from OLP tissues were first isolated in our previous study and shown to exert anti-inflammatory effects^30^, it has not been verified whether and how paeoniflorin affects the immunomodulatory function of MSCs from OLP.

In this study, we aimed to elucidate the pharmacological mechanisms underlying the therapeutic effects of paeoniflorin on MSCs and to clarify and provide experimental evidence for their clinical application. The results described here reveal that OLP-MSC-mediated immune effects may be related to regulation of the balance of Th1/Th2 cytokines in PHA-stimulated peripheral blood mononuclear cells (PBMCs) and skin graft animal models. The effects of paeoniflorin on MSCs are due to the regulation of immunomodulation via cytokines in the inflammatory microenvironment.

## Methods

### Patients

The clinical and pathological diagnosis of OLP conformed to World Health Organization diagnostic criteria^31^. A summary of each participant’s information is shown in Table 1. The study was approved by the Peking University Third Hospital Medical Science Research Ethics Committee (M2020092), and every participant provided written informed consent.

**Table 1:** The characteristics of participants recruited in the experiment.

No	Group	Gender	Age (year)	BMI	Education (year)	OLP or benign mass type	Sites of mucosal involvement
1	OLP	F	57	19.9	12	Erosion	Gingiva, Buccal mucosa, gingival buccal sulcus
2	OLP	M	62	26.4	12	Reticulate	Gingiva, Buccal mucosa
3	OLP	F	55	22.9	16	Erosion	Buccal mucosa, gingival buccal sulcus
4	OLP	M	40	21.6	16	Erosion	Lip, Buccal mucosa, Gingiva
5	OLP	F	51	19.8	12	Reticulate	Buccal mucosa, Gingiva
6	OLP	M	37	26.1	15	Reticulate	Buccal mucosa, Gingiva
7	OLP	F	43	20.5	16	Erosion	Buccal mucosa, Gingiva, Tongue
8	OLP	M	23	12.3	19	Reticulate	Lip, Tongue
9	OLP	F	21	26.8	15	Reticulate	Buccal mucosa, Lip
10	Control	F	41	19.5	15	Mucous retention cyst	Buccal mucosa
11	Control	M	65	27.7	15	Mucous retention cyst	Buccal mucosa
12	Control	F	45	23.4	12	Papilloma	Tongue
13	Control	M	43	25.8	19	Mucous retention cyst	Buccal mucosa
14	Control	F	44	23.4	9	Hemangioma	Buccal mucosa
15	Control	M	55	25.2	19	Mucous retention cyst	Lip
16	Control	F	43	24.8	15	Mucous retention cyst	Buccal mucosa
17	Control	M	20	23.3	12	Mucous retention cyst	Buccal mucosa
18	Control	F	31	19.4	19	Mucous retention cyst	Buccal mucosa
*P* value		> 0.05	> 0.05	> 0.05	> 0.05	/	/

*BMI* Body Mass Index, *OLP* oral lichen planus.

### Animals

Male CD1 and BALB/c mice (8–12 weeks) were obtained from the animal laboratory center under specific pathogen-free conditions (Vitalriver, Beijing, China). The mice were housed under a constant temperature (23 ± 2 °C) and humidity (50 ± 5%) and maintained on a 12 h/12 h light/dark cycle with free access to food and water. All procedures were performed with approval from the Biomedical Ethics Committee for Animal Use and Protection of Peking University and in accordance with the National Institutes of Health Guide for the Care and Use of Laboratory Animals and ARRIVE guidelines (https://arriveguidelines.org).

### Cell isolation and culture

OLP tissues were biopsied from the oral mucosa, and normal oral mucosal tissues adjacent to benign masses were obtained from patients who underwent surgical resection (Table 1). The collected tissues were treated with 2 mg/ml dispase (Sigma–Aldrich) to separate the subepithelial lamina propria from the epithelium. The mesenchymal tissues were then minced into 1 mm^3^ fragments and digested with type I collagenase (3 mg/ml) and dispase (4 mg/ml) for 1 h. The cell suspensions were filtered and plated on dishes in modified Eagle’s minimum essential medium (a-MEM; Gibco) with 10% fetal bovine serum (FBS; HyClone). Cells at passages 2 to 4 were used in subsequent experiments. All experiments were performed three times and repeated at least twice.

### Cell proliferation assay

MSCs from OLP lesions (OLP-MSCs) were seeded into 96-well tissue culture plates at a density of 1 × 10^3^ cells. Then, the cells were treated with various concentrations of paeoniflorin (0, 0.3, 3, 30, and 300 μM) (Ningbo Liwah Pharmaceutical Co., Ltd.) for 11 days. Cell proliferation was assessed by Cell Counting Kit-8 (CCK8; Dojindo Laboratory). An absorbance microplate reader (ELx808) was used to measure the optical density at 450 nm, and then the cell proliferation rates were calculated. To assess the potential immunomodulatory effect of paeoniflorin on MSCs, allogeneic T lymphocytes were assessed^32,33^. Human peripheral blood mononuclear cells (PBMCs) were obtained from four healthy controls and extracted via Ficoll gradient separation. Different numbers of human OLP-MSCs (2.5 × 10^3^/ml, 5 × 10^3^/ml, 1 × 10^4^/ml) were plated in 96-well plates in triplicate in a volume of 100 μl and allowed to adhere to the plates overnight. Resuspended PBMCs containing or lacking OLP-MSCs were added to the wells at a density of 2 × 10^5^/ml in the presence or absence of 10 μg/ml PHA (Sigma‒Aldrich). The OLP-MSCs and paeoniflorin-pretreated OLP-MSCs were then cultured with PHA-stimulated PBMCs at different ratios for 3 days. The influence of MSCs on the proliferation of allogeneic T lymphocytes was also assessed.

### Transwell migration assay

MSCs (1.5 × 10^5^/ml) without FBS were seeded in the upper chamber of 24-well Transwell plates (Corning Costar), and PHA-stimulated PBMCs were placed into the lower well to induce cell migration. MSCs invaded into the lower chambers containing PHA-stimulated PBMCs with 10% FBS in RPMI-1640 after 24 h. The cells were fixed with methanol for 20 min at room temperature and stained with 0.1% crystal violet, and the cells in at least five random fields were counted under a microscope at 200 × magnification.

### Multilineage differentiation in vitro

For osteogenic differentiation, 5 × 10^5^ OLP-MSCs or paeoniflorin-pretreated OLP-MSCs per well were cultured in induction medium in 6-well plates for 4 weeks, and the medium was changed every other day. The osteogenic induction medium was a-MEM containing 10% FBS with 10 nM dexamethasone, 0.1 mM L-ascorbic acid-2-phosphate, 2 mM glutamine and 10 mM β-glycerophosphate. Then, the cells were fixed with 4% formalin for 30 min and stained with 2% Alizarin Red S (Sigma‒Aldrich) to visualize osteogenic calcium deposition under a microscope (Olympus). For adipogenic induction, 4 × 10^5^ OLP-MSCs or paeoniflorin-pretreated OLP-MSCs per well were cultured in induction medium in 6-well plates for 21 days. The adipogenic induction medium was a-MEM containing 10% FBS supplemented with 1 µM dexamethasone, 60 mM indomethacin, 0.5 mM 3-isobutyl-l-methylxanthine, 10 mg/ml insulin, and 2 mM glutamine. Then, the cells were fixed with 4% formalin for 30 min, and adipogenic lipid droplets were detected by staining with 0.3% Oil Red O (Sigma‒Aldrich) and imaging under a microscope. In the neurogenic differentiation experiment, 5 × 10^4^ OLP-MSCs or paeoniflorin-pretreated OLP-MSCs were induced with 100 µM CoCl_2_ (Sigma‒Aldrich) in a-MEM containing 10% FBS for at least 3 days and fixed with 4% formalin for half an hour. They were then incubated with a primary antibody, rabbit polyclonal IgG specific for the neuronal cell-specific marker human microtubule-associated protein-2 (MAP-2, Bioworld Technology, USA), followed by a FITC-labeled secondary antibody (BD Biosciences, USA). Samples were observed with a confocal laser scanning microscope (LSM 5, Germany) according to previous studies^30^.

### Real-time qPCR

Total RNA in the plasma of each recipient mouse was extracted using TRIzol reagent (Invitrogen Life Technologies). A Revert Aid First Strand cDNA Synthesis Kit was used to perform reverse-transcription reactions (Fermentas), and cDNA was used as a template for each PCR. The primers were as follows: GAPDH forward primer, 5′-TGTGTCCGTCGTGGATCTGA-3′ and reverse primer, 5′-TTGCTGTTGAAGTCGCAGGAG-3′; OPN forward primer, 5′- AGCCATGAGTCAAGTCAGCT -3′ and reverse primer, 5′- ACTCGCCTGACTGTCGATAG -3′; and LPL forward primer, 5′- AGCTGACCAGTTATGGCACC-3′ and reverse primer, 5′- ATCCTGACCCTCGTAGCCTT-3′. PCR was performed using a 7500 Real-time PCR system (Applied Biosystems).

### Flow cytometry analysis

PHA-stimulated PBMCs treated with OLP-MSCs or paeoniflorin-pretreated MSCs and PHA-stimulated PBMCs were subjected to flow cytometry (BeckmanCoulter, Fullerton, CA). Cell cycle distributions were analyzed as the percentages of cells in the G0, G1, S, and G2 M phases.

### Enzyme-linked immunosorbent assay (ELISA)

OLP-MSCs or paeoniflorin-pretreated MSCs and MLR cocultures were seeded into 96-well plates at ratios of 1:20 for 3 days. Then, Th1 cytokines (TNF-α, IFN-γ, IL-1β and IL-2) and Th2 cytokines (IL-4, IL-5, IL-10 and IL-13) released into the supernatants were collected for assessment by ELISA kits (Proteintech) according to the manufacturer’s instructions.

### EdU labeling of OLP-MSCs

EdU was used to label cells for transplantation in vivo. OLP-MSCs were seeded into a 10-cm dish in medium with 20 μM EdU (Invitrogen, Carlsbad, CA) to label the cells. Twenty-four hours later, the cells were washed with PBS 3 times, added to the culture medium for further incubation and prepared for in vivo transplantation. Previous studies have shown that coinfusion of MSCs with unmodified donor bone marrow improves vascularized skin graft survival, indicating the potential for the development of autologous MSC-based bone marrow transplantation and the prevention of graft rejection^34^. Moreover, in a xenogeneic GVHD NOD/Shi-scid IL2rγnull mouse model, human amnion-derived MSCs induced marked immunosuppression and delayed acute GVHD progression^35^.

### MSC transplantation in vivo

Male BALB/c (8–12 weeks old) recipients were divided randomly into 4 groups (n = 6 per group), and CD1 mice served as donors. Skin grafts (15 mm^2^) obtained from the backs of donors were transplanted into recipients, and 2 × 10^6^ MSCs were injected into the recipients. The groups were divided into the following groups: (1) the control group (no transplantation); (2) the skin graft group (a naive group without any treatment); (3) the OLP-MSC injection group; and (4) the paeoniflorin-pretreated OLP-MSC injection group. A saline injection group was used as a negative control.

Skin rejection was observed and recorded from the third day after transplantation. Rejection was defined as eschar formation or epidermal sloughing and served as an indicator of the graft survival time. Mouse survival data were collected for each group 3 weeks after transplantation, and punch biopsies were fixed in neutral-buffered formalin for HE on day 14. Skin rejection after transplantation was evaluated, and each slide was given a histological score ranging from 1 to 5 according to previously reported parameters^36^. The serum of recipient mice was harvested before surgery. The expression levels of Th1 cytokines (TNF-α, IFN-γ, IL-1β and IL-2) and Th2 cytokines (IL-4, IL-5, IL-10 and IL-13) were measured using ELISA.

### Tracking of transplanted OLP-MSCs

After EDU-labeled MSC transplantation, tissues were harvested, fixed and embedded in paraffin on day 1. Then, 4 mm sections were prepared and washed three times with PBS. Subsequently, the sections were incubated in 3% bovine serum albumin (BSA) in PBS, followed by 0.5% Triton® X-100 in PBS for 20 min. Freshly prepared iClick reaction cocktails, which contained 1 × iClick reaction buffer, CuSO_4_, Andy Flour 647 azide, and 1 × reaction buffer additive (iClick™ EDU Andy Fluor 647 Imaging Kit, GeneCopoeia™), were incubated with tissues for 30 min in the absence of light at room temperature. The reaction cocktail was removed, and the tissues were washed with PBS. Nuclei were stained with DAPI for 5 min, and the tissues were washed twice with PBS and imaged by fluorescence microscopy. At least three fields were randomly captured, and the percentage of positive staining was measured with ImageJ software (National Institute of Health, USA. URL: http://imagej.nih.gov/ij).

### Statistical analysis

Data analyses were performed by SPSS 26.0 and are presented as the mean ± SEM. One-way analysis of variance (ANOVA) and post hoc Bonferroni tests were used to analyze differences among three or more groups. Survival curves were plotted by the Kaplan‒Meier method, and log-rank analysis was used to compare the survival rates between groups. *P* < 0.05 was considered statistically significant.

## Results

### Paeoniflorin promotes MSC proliferation and migration in vitro

**Figure 1:**
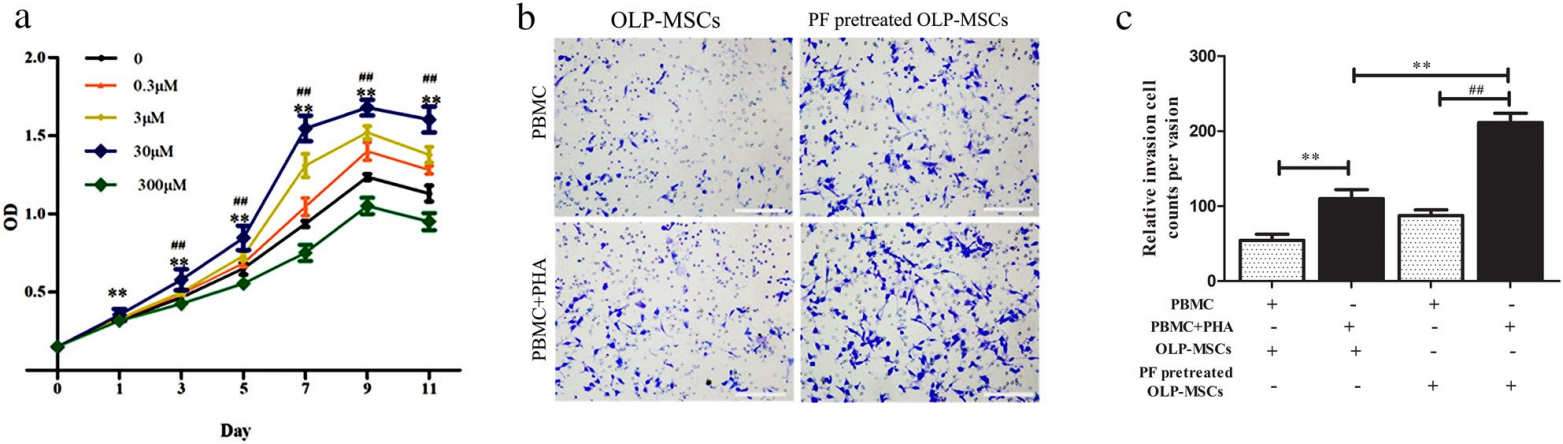
Characteristics of OLP-MSCs and paeoniflorin-pretreated OLP-MSCs. (**a**) Comparison of cell proliferation capacities. The proliferative capacity of OLP-MSCs was increased by paeoniflorin. The ability of MSCs to invade T lymphocytes was assessed by a migration assay. (**b**) Representative invasion images of paeoniflorin-pretreated OLP-MSCs. (**c**) Relative invasion of paeoniflorin-pretreated OLP-MSCs. Scale bars, 100 μm. All data are expressed as the means ± SEMs. **P < 0.01 compared with d 0; ##*P* < 0.01, compared with OLP-MSCs on the same day. PF, paeoniflorin.

To investigate the effects of paeoniflorin on the proliferation of MSCs, different concentrations of paeoniflorin were added to OLP-MSCs for 11 days. We found that the effects of paeoniflorin on the proliferation of MSCs were time- and dose dependent. The results showed that paeoniflorin could promote the proliferation of OLP-MSCs, and the effect of the 30 μM paeoniflorin treatment group was better than that of the other groups and was significantly different from the effect of OLP-MSCs from the 3rd day (Fig. 1a, *P* < 0.05), with a peak on the 9th day. We next investigated the migration potential of OLP-MSCs after paeoniflorin pretreatment in Transwell cultures. There was a significant difference in the number of transmigrated OLP-MSCs between the groups cultured in the absence or presence of 30 μM paeoniflorin pretreatment (109.67 ± 12.17% versus 211.33 ± 12.35% after paeoniflorin pretreatment) at 24 h (Fig. 1b and c, *P* < 0.01). Paeoniflorin pretreatment improved OLP-MSC migration in a Transwell assay in vitro.

### Paeoniflorin promotes MSC multilineage differentiation

**Figure 2:**
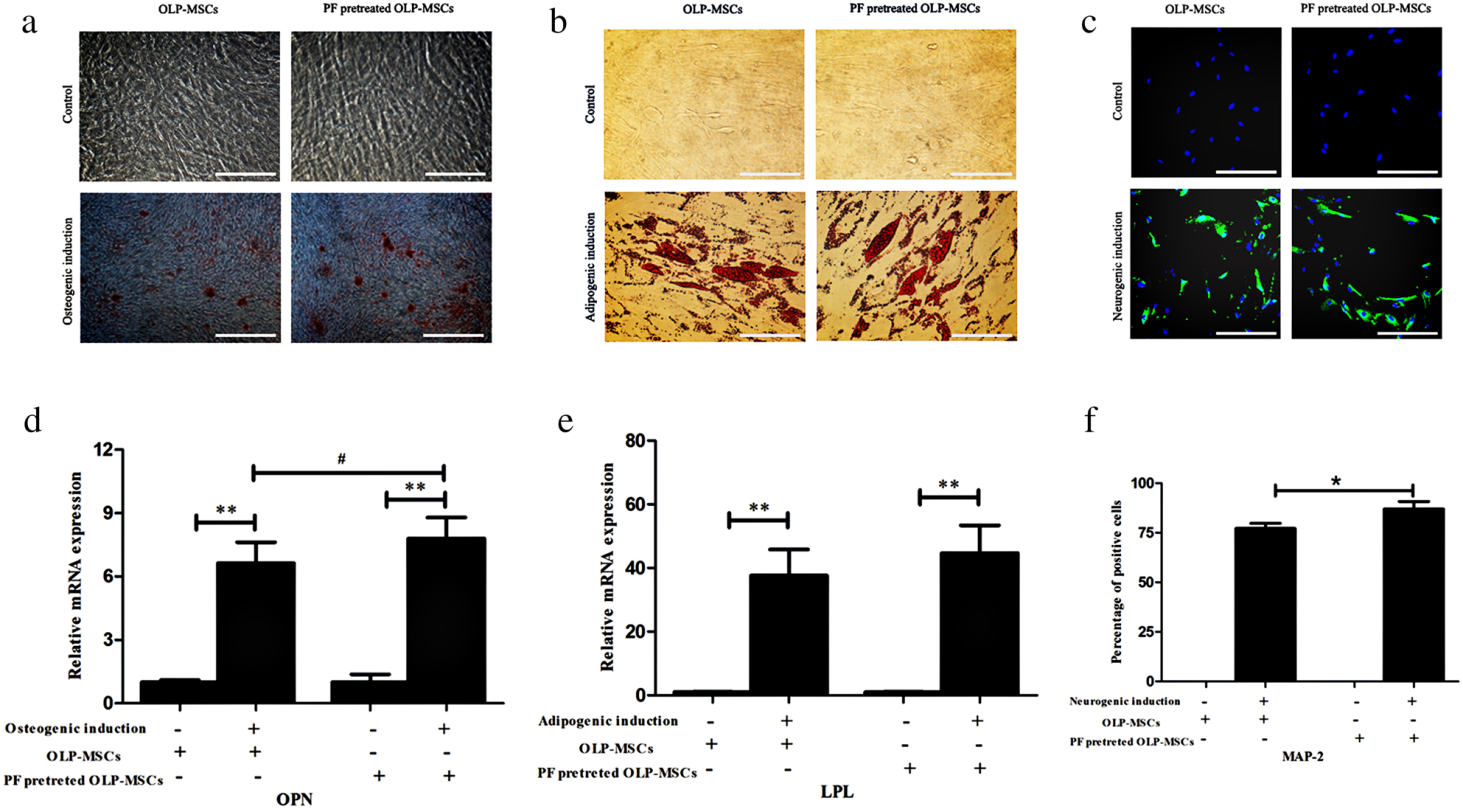
Multipotent differentiation of paeoniflorin-pretreated OLP-MSCs. (**a**) Osteogenic differentiation potential of OLP-MSCs and paeoniflorin-pretreated OLP-MSCs. Mineralized nodules were observed in the induced groups, as assessed by alizarin red staining, and were not formed in the control groups. (**b**) Adipogenic differentiation potential of OLP-MSCs and paeoniflorin-pretreated OLP-MSCs. Oil red O staining of accumulated lipid droplets was performed in the induced cells and was not observed in the control cells. (**c**) Neurogenic differentiation potential of OLP-MSCs and paeoniflorin-pretreated OLP-MSCs. The morphology of MSC neural changes after immunofluorescence staining for neuron-specific enolase (MAP-2); these changes were not found in the control groups. (**d**) Analysis of OPN mRNA expression during osteogenic differentiation, as assessed by real-time PCR (n > 4). (**e**) Analysis of LPL mRNA expression during adipogenic differentiation, as assessed by real-time PCR (n > 4). (**f**) Semiquantification of MAP-2-positive cells after immunofluorescence staining (n > 4). Scale bar = 100 μm. All data are expressed as the means ± SEMs. *P < 0.05, **P < 0.01; #*P* < 0.05. PF, paeoniflorin.

MSCs from OLP maintain multidifferentiation capacity. In osteogenic differentiation studies, we found that osteogenesis induction in paeoniflorin-pretreated OLP-MSC was improved compared with that in OLP-MSCs (Fig. 2a and d, *P* < 0.05). In the adipogenic differentiation analysis, there was no difference between induced paeoniflorin-pretreated OLP-MSCs and OLP-MSCs (Fig. 2b and e). After neurogenic differentiation, the percentage of neuronal cells in paeoniflorin-pretreated OLP-MSCs was increased compared with that in OLP-MSCs, as illustrated by the neuron-specific marker MAP-2 and indirect immunofluorescence analysis (Fig. 2c and f, *P* < 0.05).

### Paeoniflorin-pretreated MSCs suppress the proliferation of PBMCs

**Figure 3:**
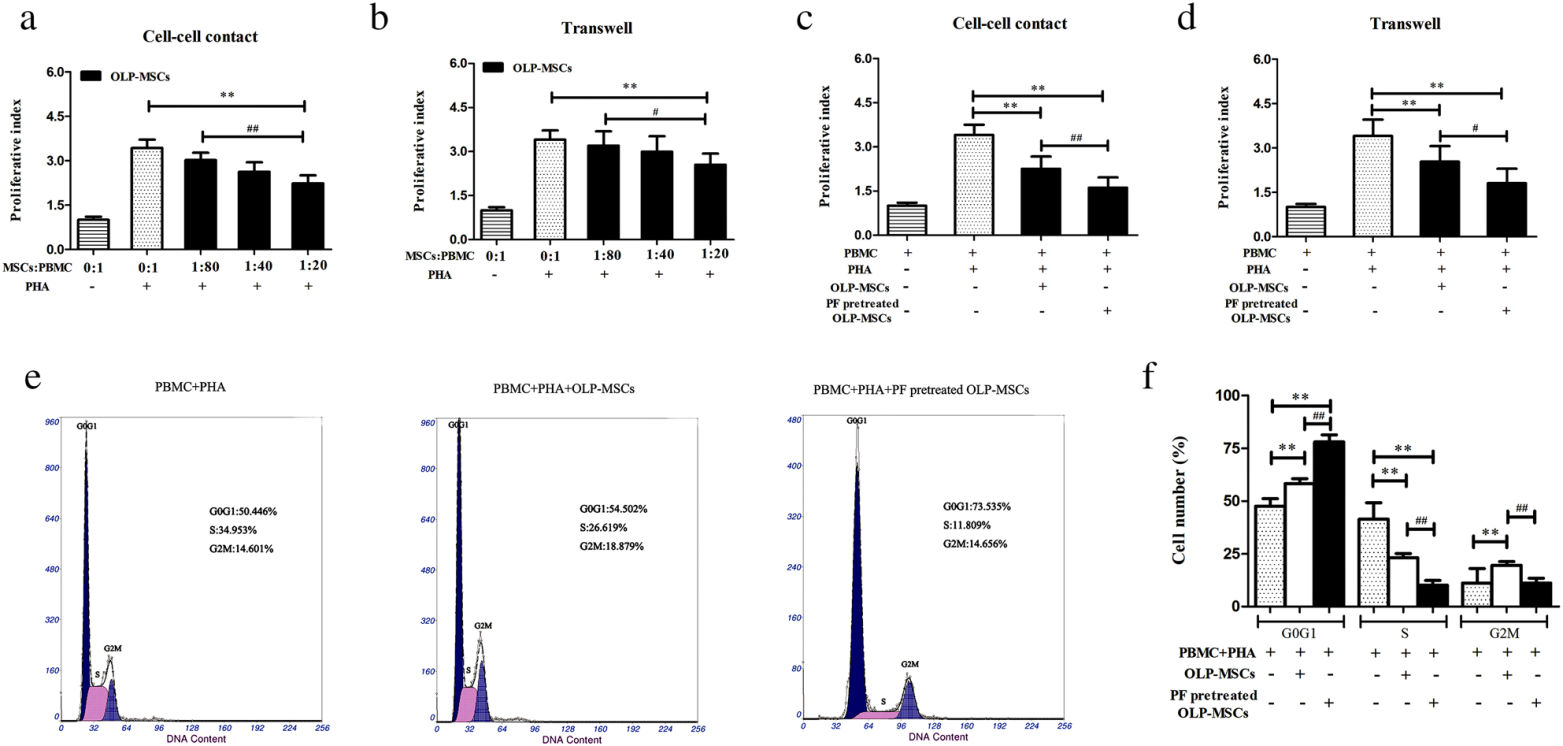
Inhibitory effects of OLP-MSCs and paeoniflorin-pretreated OLP-MSCs on PHA-stimulated PBMC proliferation. PBMCs were cultured alone or cocultured with increasing numbers of OLP-MSCs or paeoniflorin-pretreated OLP-MSCs in the presence or absence of PHA for 72 h. Afterward, cell numbers were counted using a Cell Counting Kit-8. The most effective ratio for the suppression of allogeneic T-cell proliferation was 1:20 under (**a**) cell‒cell contact and (**b**) Transwell conditions. Analysis of the T-cell proliferation rate after paeoniflorin pretreatment of MSCs under (**c**) cell‒cell contact and (**d**) Transwell conditions. The proliferation effect of MSCs on T lymphocytes was assessed by a Cell Counting Kit-8. (**e**) Effect of OLP-MSCs and paeoniflorin-pretreated OLP-MSCs on T lymphocyte cell cycle progression by flow cytometry. (**f**) Representative flow cytometry graphs of cells treated with OLP-MSCs and paeoniflorin-pretreated OLP-MSCs for 3 days (top). Mean values of the cell cycle distribution in different groups over 3 days (n > 4, *P < 0.05, **P < 0.01; #*P* < 0.05, ##*P* < 0.01). PF, paeoniflorin.

Next, we sought to determine whether paeoniflorin influences the immunosuppressive effects of MSCs on the proliferation of T lymphocytes in vitro. To this end, OLP-MSCs were cocultured with human PBMCs under cell‒cell contact or Transwell systems for 3 days. Our results showed that OLP-MSCs inhibited PHA-stimulated PBMC proliferation in both cell‒cell contact and Transwell cultures in a cell density-dependent manner. The OLP-MSC-mediated inhibition of T lymphocyte proliferation was stronger under cell‒cell contact conditions than in Transwell plates (Fig. 3a and b). In addition, the inhibition of proliferation in T cells cocultured with paeoniflorin-pretreated OLP-MSCs was significantly enhanced under both cell‒cell contact and Transwell conditions (Fig. 3c and d, *P* < *0.05*). Meanwhile, our data also indicated that paeoniflorin-pretreated OLP-MSCs inhibited the proliferation of T lymphocytes more strongly under cell‒cell contact conditions than in Transwell plates (Fig. 3c and d). These results suggest that direct cell‒cell contact contributes, at least in part, to the mechanisms by which paeoniflorin-pretreated OLP-MSCs mediate immunosuppression via suppression of T lymphocyte proliferation. We further investigated the possible effects of paeoniflorin-pretreated MSCs on the regulation of cell cycle progression in PBMCs (Fig. 3e). The data showed that a higher percentage of cells in G0 G1 phase (58.2% versus 47.5%) and a lower percentage of cells in S phase (23.1% versus 41.4%) were detected in PHA-stimulated PBMCs treated with OLP-MSCs than in untreated cells (Fig. 3f, *P* < 0.05). In the paeoniflorin-pretreated OLP-MSC-treated PBMC group, most cells remained in the G0 G1 phase (78%), and few cells were in S phase (10.1%). Combined with the CCK-8 results, the flow cytometry results suggested that paeoniflorin-pretreated OLP-MSCs inhibited the growth of PBMCs through G1 phase cell cycle arrest and a decrease in the number of cells in S phase.

### Paeoniflorin-pretreated MSCs regulate cytokines in cocultures

**Figure 4:**
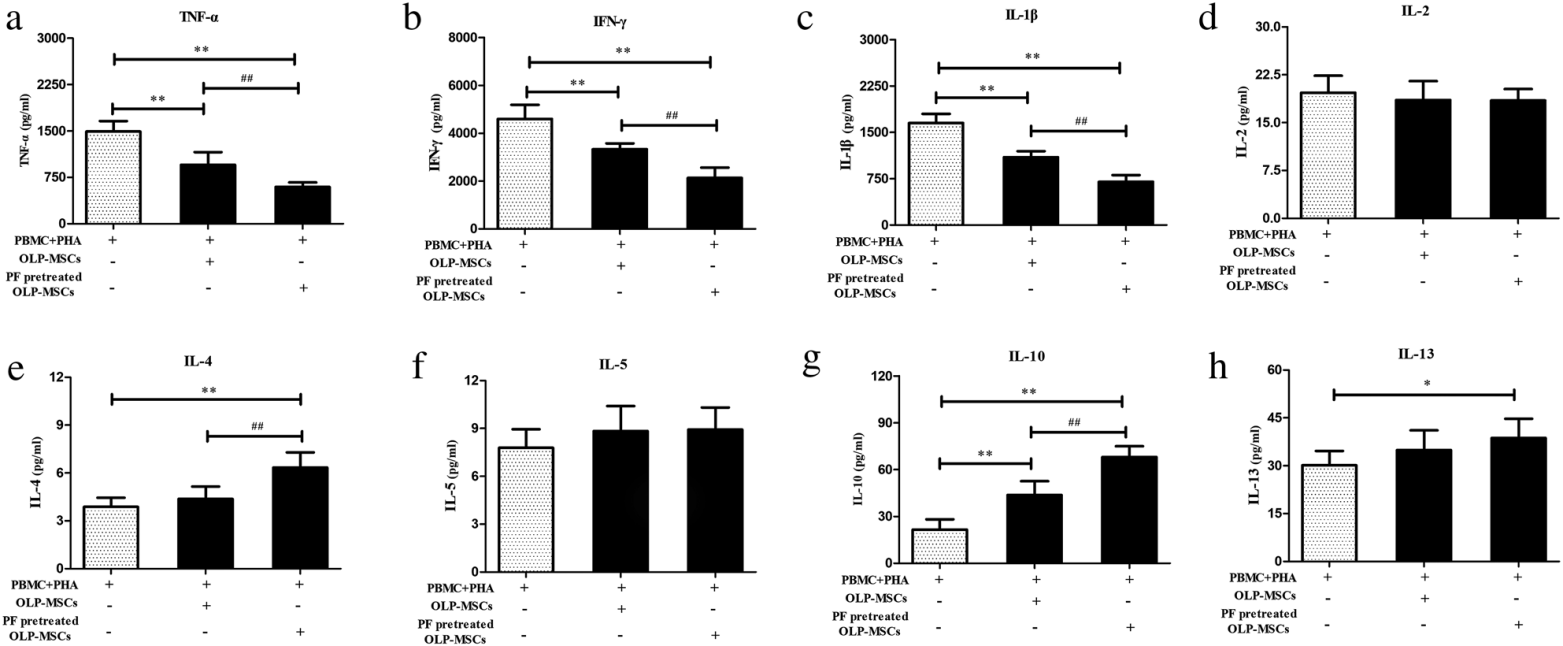
The production of immunomodulatory cytokines in T cells cocultured with OLP-MSCs and paeoniflorin-pretreated OLP-MSCs. Small amounts of the Th1 cytokines (**a**) TNF-α, (**b**) IFN-γ, (**c**) IL-1β and (**d**) IL-2 were produced, while large amounts of the Th2 cytokines (**e**) IL-4, (**f**) IL-5, (**g**) IL-10 and (**h**) IL-13 were produced by T cells cocultured with OLP-MSCs or paeoniflorin-pretreated OLP-MSCs using ELISA. The regulatory ability of paeoniflorin-pretreated OLP-MSCs was stronger than that of the untreated MSCs (*P < 0.05, **P < 0.01; #*P* < 0.05, ##*P* < 0.01). PF, paeoniflorin.

We next determined the role of soluble mediators in MSC-mediated immunosuppression of T lymphocytes. To this end, we measured cytokine secretion in MSC and PBMC cocultures under cell‒cell contact conditions. The levels of the Th1 cytokines TNF-α (Fig. 4a, *P* < 0.01), IFN-γ (Fig. 4b, *P* < 0.01) and IL-1β (Fig. 4c, *P* < 0.01) were decreased, while the level of IL-2 was not changed (Fig. 4d, *P* > 0.05). The levels of the Th2 cytokines IL-4 (Fig. 4e, *P* < 0.01), IL-10 (Fig. 4g, *P* < 0.01) and IL-13 (Fig. 4h, *P* < 0.05) but not IL-5 (Fig. 4f, *P* > 0.05) were increased in the supernatant of T lymphocytes cocultured with OLP-MSCs. Paeoniflorin-pretreated OLP-MSCs exacerbated the changes in the secretion of these cytokines. Taken together, these results suggest that paeoniflorin-pretreated MSCs are capable of affecting the secretion of cytokines after activation of T lymphocytes in cocultures.

### Tracking of transplanted MSCs

**Figure 5:**
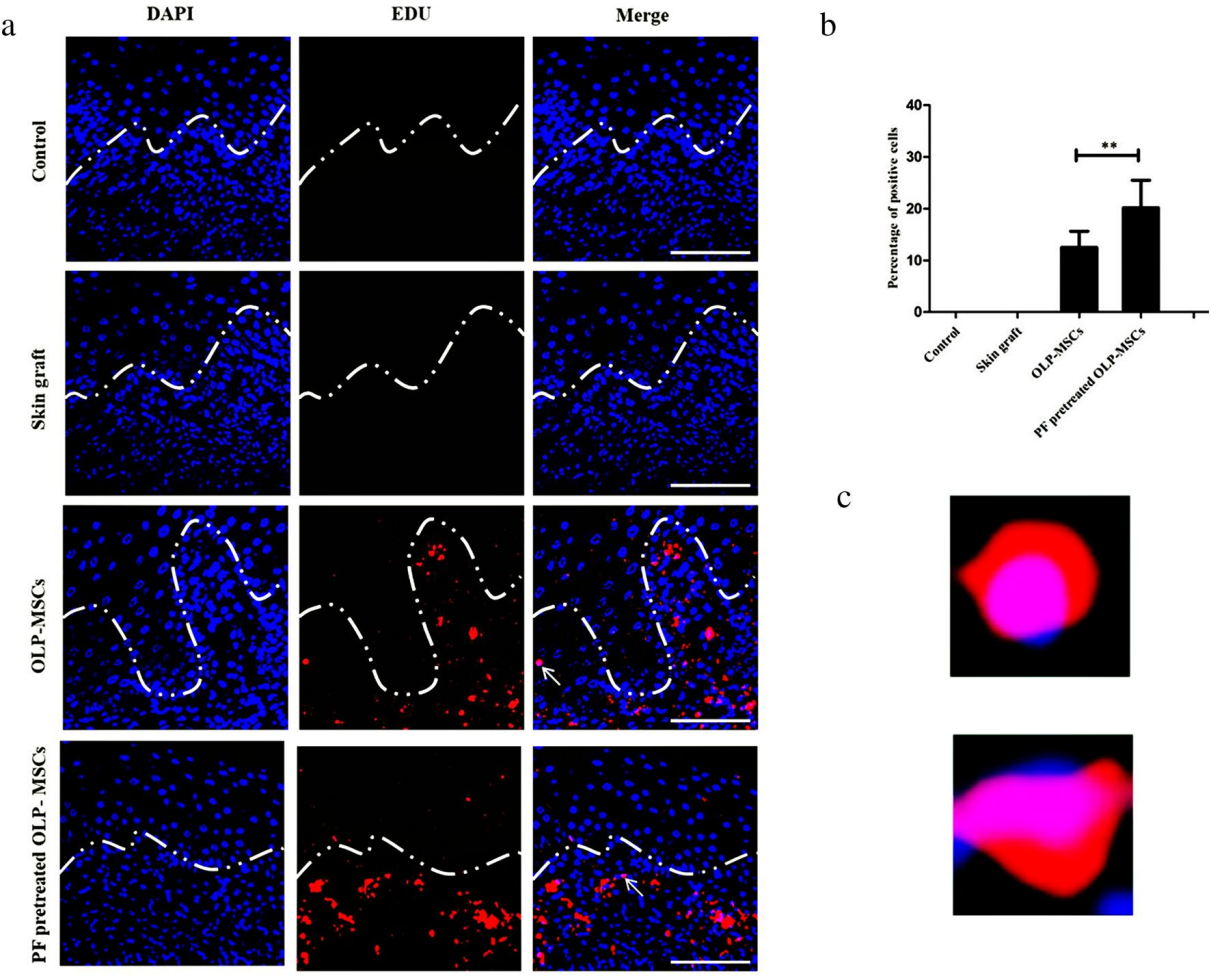
EdU-labeled OLP-MSCs and paeoniflorin-pretreated OLP-MSCs in skin tissues. Tissues were harvested on the 3rd day and stained with EdU (red fluorescence) and DAPI (blue fluorescence). (**a**) The EdU- and DAPI-stained images were digitally merged. Four representative tissues at 400 × magnification. (**b**) Quantification of EdU-labeled OLP-MSCs and paeoniflorin-pretreated OLP-MSCs. (**c**) Magnified images of the nuclei indicated by arrowheads in the graphs in panel (**a**). Scale bar = 50 μm. **P < 0.01. PF, paeoniflorin.

To demonstrate that MSCs can migrate to sites of inflammation and injury in vivo, EdU-labeled OLP-MSCs and paeoniflorin-pretreated OLP-MSCs were transplanted intravenously into mice that suffered skin graft rejection. On day 3 after transplantation, MSCs were visible in the skin graft by immunofluorescence staining with EDU (Fig. 5a and c). The average numbers of EdU-positive MSCs in the skin per field at three time points were 12.51 ± 3.11 and 20.17 ± 5.33 in the OLP-MSC and paeoniflorin-pretreated OLP-MSC groups, respectively (Fig. 5b, *P* < 0.01).

### Paeoniflorin-pretreated MSC therapy in mice with skin grafts

**Figure 6:**
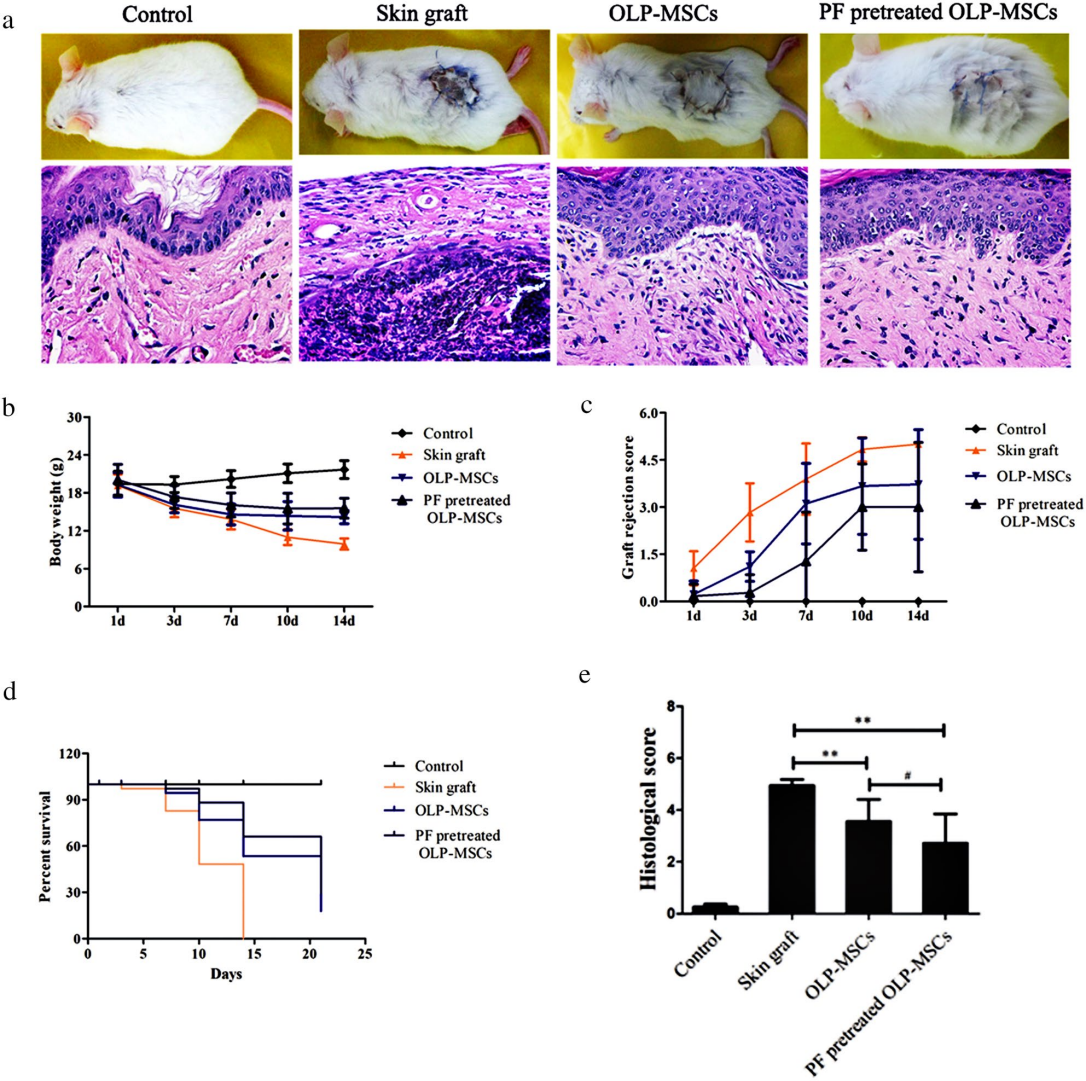
Treatment with paeoniflorin-pretreated OLP-MSCs and paeoniflorin-pretreated OLP-MSCs ameliorated experimental skin graft rejection in mice. Paeoniflorin-pretreated MSCs prolonged skin graft survival and reduced the inflammatory response. MSCs (2 × 10^6^) were injected into the recipients. (**a**) Skin graft changes and histological HE staining, (**b**) body weight changes, (**c**) graft rejection scores, (**d**) a Kaplan–Meier curve of survival time and (**e**) histological scores showed that OLP-MSCs prolonged the survival time of the skin graft and inhibited the infiltration of lymphocytes. Paeoniflorin-pretreated OLP-MSCs showed improved immunomodulatory function in animals that received skin grafts (**P < 0.01; #*P* < 0.05). PF, paeoniflorin.

To further explore the potential therapeutic effects of OLP-MSCs or paeoniflorin-pretreated OLP-MSCs, the cells were infused into an established murine skin graft model to test whether they could reverse tissue injury and control inflammation. After skin transplantation, graft rejection was detected, similar to a previous report^37^. The group treated with MSCs had less rejection, and paeoniflorin improved the ability of OLP-MSCs to alleviate graft rejection (Fig. 6a). Compared with the skin graft group, the OLP-MSC group showed reduced infiltration of inflammatory cells. The inhibitory effects of paeoniflorin-pretreated OLP-MSCs on inflammatory infiltration yielded improved immune regulation (Fig. 6a and e, *P* < 0.05).

All mice died 14 days after skin grafting without MSC transplantation. We confirmed the presence of sustained weight loss in the group with skin graft rejection. OLP-MSC application alleviated this change, and the paeoniflorin-pretreated MSC groups showed a slight reduction in weight loss (Fig. 6b). Compared with the skin graft group, the OLP-MSC group showed prolonged skin graft survival time, and the paeoniflorin pretreated OLP-MSC group showed an improved graft rejection score, which was superior to that of the untreated group (Fig. 6c). The survival rates of OLP-MSCs and paeoniflorin-pretreated OLP-MSCs were 53.44% and 66.17%, respectively (Fig. 6d).

### Immunosuppressive activity of paeoniflorin-pretreated MSCs in vivo

**Figure 7:**
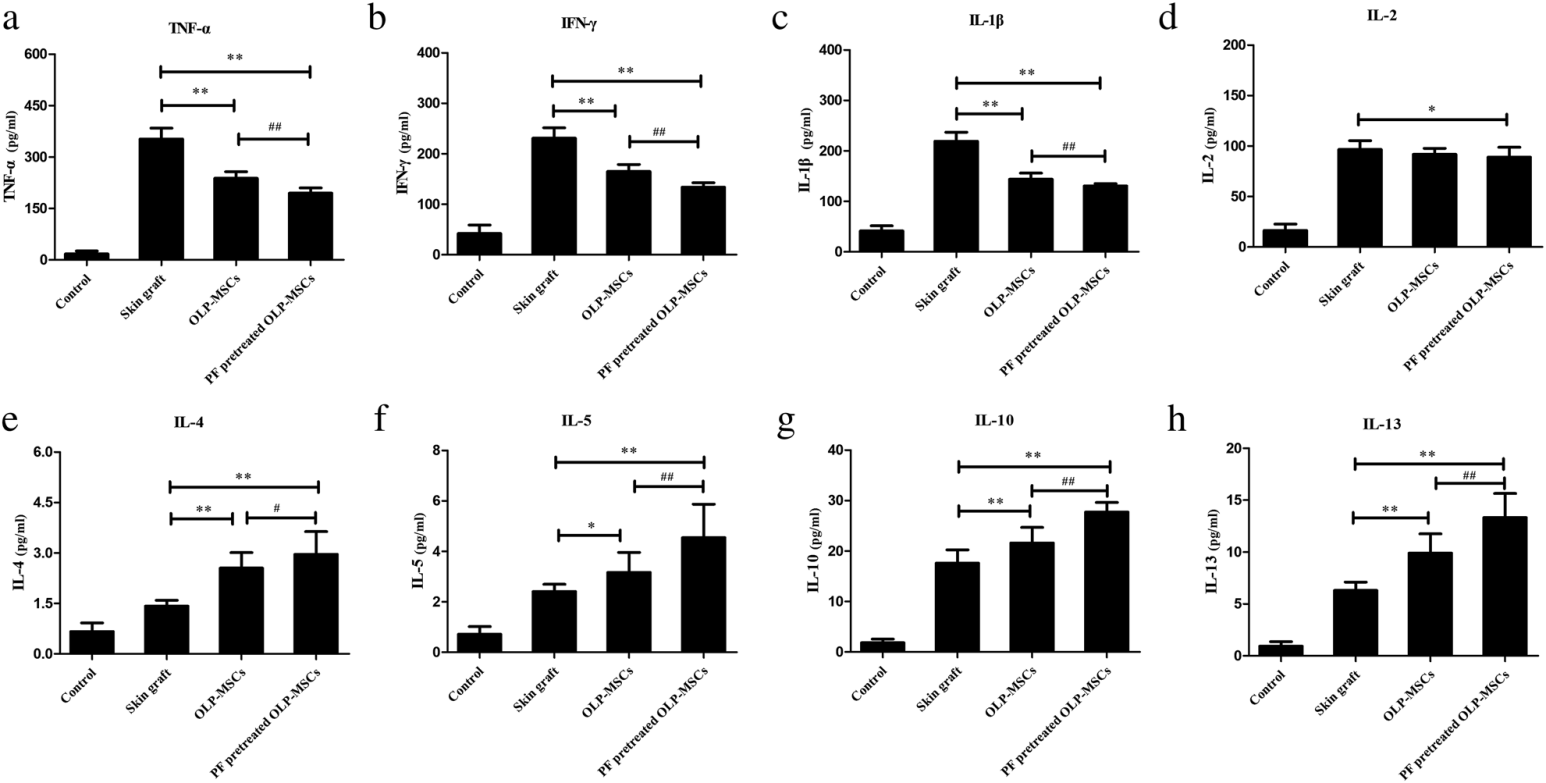
Serum cytokine levels in mice that received skin allografts. Small amounts of the Th1 cytokines (**a**) TNF-α, (**b**) IFN-γ, (**c**) IL-1β and (**d**) IL-2 and large amounts of the Th2 cytokines (**e**) IL-4, (**f**) IL-5, (**g**) IL-10 and (**h**) IL-13 were produced in the OLP-MSC injection group, as shown by ELISA. Paeoniflorin pretreatment improved the anti-inflammatory effects of OLP-MSCs in mice subjected to skin transplantation (**P < 0.01; #*P* < 0.05, ##*P* < 0.01). PF, paeoniflorin.

The levels of the Th1 cytokines TNF-α (Fig. 7a, *P* < 0.01), IFN-γ (Fig. 7b, *P* < 0.01), IL-1β (Fig. 7c, *P* < 0.01) and IL-2 (Fig. 7d, *P* < 0.05) were decreased, and the levels of the Th2 cytokines IL-4 (Fig. 7e, *P* < 0.05), IL-5 (Fig. 7f, *P* < 0.01), IL-10 (Fig. 7g, *P* < 0.05) and IL-13 (Fig. 7h, *P* < 0.05) were increased in the serum of mice that received skin allografts after OLP-MSC treatment. Compared with MSCs alone, paeoniflorin-pretreated OLP-MSCs exacerbated the changes in the secretion of these cytokines. These results suggest that paeoniflorin could enhance the anti-inflammatory properties of MSCs and reduce immune rejection in mice that received allogeneic skin grafts.

## Discussion

The results of the present study indicate that paeoniflorin enhanced MSC immunomodulation and regulated the inflammatory microenvironment via Th1/Th2 cytokine production by T lymphocytes. Specifically, paeoniflorin-pretreated OLP-MSCs exacerbated the changes in the secretion of these cytokines. OLP-MSCs prolonged the skin graft survival time and improved the graft rejection scores and survival rates. Additionally, paeoniflorin-pretreated OLP-MSCs altered the Th1/Th2 balance in the serum of mice that received skin allografts, prolonged skin graft survival and ameliorated inflammatory infiltration in an experimental mouse model.

A new population of precursor cells from the normal human oral mucosa and OLP lesions, termed OMMSCs and OLP-MSCs, has been isolated and characterized; these cells exhibited several unique stem cell-like properties similar to those of MSCs derived from bone marrow in our previous research^26^. MSCs are a population of adult multipotent stem or stromal cells that differentiate into osteocytes, adipocytes, neuroectodermal progenies, have proliferation and colony-forming abilities in vitro, and express MSC surface markers^16,38^. MSCs migrate and proliferate within damaged, inflamed and malignant tissues as part of the tissue regeneration process, and they also display immunomodulatory properties^39,40^. These unique characteristics make MSCs attractive candidates for cell-based therapeutic strategies fr the repair and regeneration of inflamed or damaged tissues^41^.

As previous studies have reported, MSCs display chemotactic properties in response to inflammation and tissue insult, thus exhibiting a tendency to migrate toward the site of inflammation^42,43^. Proinflammatory cytokines can modulate MSC behaviors in situ in inflamed and injured tissues^44^. OLP is a chronic oral mucosal inflammatory disease mediated by T cells, and simultaneous expression of Th1 and Th2 cytokines arises in local OLP lesions and tissue transudates^45,46^. This evidence indicates that the function of MSCs may be affected by the inflammatory microenvironment in OLP. Nevertheless, OLP-MSCs still have the ability to induce inflammation via chemotaxis, while paeoniflorin increases the migration ability of OLP-MSCs.

Our previous study showed that the expression of the inflammatory cytokines IFN-γ, IL-6, TNF-α, and IL-10 was increased in OLP tissues compared with normal subepithelial lamina tissues^30,47^. The ratio of the Th1 cytokine IFN-γ to the Th2 cytokine IL-4 in OLP patients increased significantly, and a Th1 cytokine predominance was proven^8^. Hence, the balance of Th1/Th2 cytokines plays an important role in the pathogenesis and progression of OLP due to immunological regulation^48^. Here, we found that OLP-MSCs have anti-inflammatory effects via the reduction of Th1 cytokine levels (TNF-α, IFN-γ and IL-1β) and elevation of Th2 cytokine levels (IL-10 and IL-13) through direct cell‒cell contact in inflammatory conditions, which may be one of the key mechanisms of MSC immunomodulatory effects in OLP. MSCs could induce cocultured macrophages to produce increased IL-10 and decreased TNF-α levels at higher temperatures^18^. Therefore, MSC-mediated immune responses in OLP may be related to reversing the imbalance of Th1/Th2 and immunosuppressive factors.

The main treatment for OLP involves the administration of topical or systemic drugs^49^. TGP is a natural traditional Chinese medicine and has curative effects for OLP without toxicity to the liver, kidney, or nervous system^50^. TGP inhibits Th1/Th17 cells by decreasing the maturation and activation of dendritic cells in rheumatoid arthritis^51^. The levels of the Th1 cytokines IL-12, IFN-γ and TNF-α significantly decreased during TGP treatment in psoriatic arthritis^52^. TGP inhibited LPS-induced production of IL-6 and TNF-α and simultaneously decreased the phosphorylation of IκBα and NF-κB p65 in HaCaT cells^53^. As the main component of TGP, paeoniflorin not only inhibits inflammation by regulating cytokines but also improves the inhibition of T lymphocyte proliferation induced by OLP-MSCs. Paeoniflorin increased the proliferation and regulatory properties of OLP-MSCs, which may be helpful for further understanding the crucial mechanisms of action of TGP pharmacological treatment in OLP.

MSCs have exhibited early efficacy in attenuating the progression of several experimental inflammatory diseases. Recently, anti-inflammatory or immunomodulatory properties, trophic effects on tissue repair and homing to sites of inflammation have made MSCs popular treatments in murine models and clinical trials^54^. The positive treatment effects of human OLP-MSCs on graft rejection could be due to their inherent ability to regulate immune tolerance by increasing the production of anti-inflammatory cytokines and controlling inflammatory infiltration, which is consistent with previous studies^55,56^. Paeoniflorin has been used to protect against liver ischemia/reperfusion injury by inhibiting the HMGB1-TLR4 signaling pathway^57^. Notably, paeoniflorin can significantly improve the anti-inflammatory effects of MSCs by regulating the proportion of cytokines, suggesting a potential pharmacological therapy involving the application of MSCs. It is also worth noting that several studies have shown that autoimmune factors, especially desmoglein 3, might be involved in the etiology of OLP. However, the findings are inconsistent. For example, one case‒control study showed that anti-desmoglein 3 antibody levels are likely to be elevated in erosive OLP but not in other types of OLP^58^, while another study revealed that unclassified OLP is associated with anti-desmoglein 3 autoantibodies^59^. Since it is unclear whether the function of anti-desmoglein 3 antibodies is altered in patients with OLP, further clarifications are required to elucidate the etiology of this disease.

Based on our observations, paeoniflorin enhanced MSC immunomodulation and regulated the inflammatory microenvironment via T lymphocytes in mice that received skin grafts. Moreover, as expected, the efficacy of paeoniflorin-treated MSCs was improved, suggesting that activated MSCs can be applied as a novel cell source for clinical cell-based treatment of immune-mediated inflammatory diseases.

The limitations of this study include the fact that although we have demonstrated that paeoniflorin, the main component of TGP, exerts immune effects in OLP by regulating MSCs, the amount of paeoniflorin in tissue is limited, and its regulatory mechanism is not completely clear. In addition, further investigations should be conducted to establish a universally recognized animal model that better reflects the precancerous immune inflammatory response of OLP. These clarifications will be helpful for improving the understanding of the pathogenesis and prevention of oral carcinoma.

## Conclusions

Our study showed that paeoniflorin promotes the proliferation, migration and multilineage differentiation of MSCs from OLP lesions via regulation of the Th1/Th2 balance to prolong skin graft survival and ameliorate inflammatory infiltration. Overall, paeoniflorin enhances MSC immunomodulation and regulates the inflammatory microenvironment via T lymphocytes, providing a promising therapeutic strategy for OLP treatment through improvement of MSC function.

## Data Availability

The datasets used and/or analysed during the current study available from the corresponding author on reasonable request.

## Abbreviations

LP: Lichen planus
OLP: Oral lichen planus
MSCs: Mesenchymal stem cells
PBMC: Peripheral blood mononuclear cell
PF: Paeoniflorin
ELISA: Enzyme-linked immunosorbent assay
OLP-MSCs: MSCs from OLP lesions
